# CMR left ventricular strains beyond global longitudinal strain in differentiating light-chain cardiac amyloidosis from hypertrophic cardiomyopathy

**DOI:** 10.3389/fcvm.2023.1108408

**Published:** 2023-05-03

**Authors:** Fangqing Wang, Yan Deng, Shunjia Li, Qichao Cheng, Qing Wang, Dexin Yu, Qian Wang

**Affiliations:** ^1^ Department of Radiology, Qilu Hospital, Shandong University, Jinan, China; ^2^ Department of Radiation Oncology, Qilu Hospital, Shandong University, Jinan, China

**Keywords:** cardiovascular magnetic resonance, light-chain cardiac amyloidosis, hypertrophic cardiomyopathy, left ventricular function, global longitudinal strain, long-axis strain

## Abstract

**Background:**

The clinical value of left ventricular (LV) global longitudinal strain (GLS) in the differential diagnosis of light-chain cardiac amyloidosis (AL-CA) and hypertrophic cardiomyopathy (HCM) has been previously reported. In this study, we analyzed the potential clinical value of the LV long-axis strain (LAS) to discriminate AL-CA from HCM. Furthermore, we analyzed the association between all the LV global strain parameters derived from cardiac magnetic resonance (CMR) feature tracking and LAS in both the AL-CA and HCM patients to assess the differential diagnostic efficacies of these global peak systolic strains.

**Materials and methods:**

Thus, this study enrolled 89 participants who underwent cardiac MRI (CMRI), consisting of 30 AL-CA patients, 30 HCM patients, and 29 healthy controls. The intra- and inter-observer reproducibility of the LV strain parameters including GLS, global circumferential strain (GCS), global radial strain (GRS), and LAS were assessed in all the groups and compared. Receiver operating characteristic (ROC) curve analysis was performed to determine the diagnostic performances of the CMR strain parameters in discriminating AL-CA from HCM.

**Results:**

The intra- and inter-observer reproducibility of the LV global strains and LAS were excellent (range of interclass correlation coefficients: 0.907–0.965). ROC curve analyses showed that the differential diagnostic performances of the global strains in discriminating AL-CA from HCM were good to excellent (GRS, AUC = 0.921; GCS, AUC = 0.914; GLS, AUC = 0.832). Furthermore, among all the strain parameters analyzed, LAS showed the highest diagnostic efficacy in differentiating between AL-CA and HCM (AUC = 0.962).

**Conclusion:**

CMRI-derived strain parameters such as GLS, LAS, GRS, and GCS are promising diagnostic indicators that distinguish AL-CA from HCM with high accuracy. LAS showed the highest diagnostic accuracy among all the strain parameters.

## Introduction

Both light-chain cardiac amyloidosis (AL-CA) and hypertrophic cardiomyopathy (HCM) cause myocardial hypertrophy and left ventricular (LV) dysfunction and are often misdiagnosed in the clinic (1). However, the underlying pathogenetic mechanisms of AL-CA and HCM are significantly different. In AL-CA, the deposition of abnormal light-chain proteins in the extracellular matrix of the myocardial tissue interferes with cell–cell coupling and disrupts cellular integrity; moreover, deposition of the light-chain fibers increased oxidative stress, which subsequently damages the proteins involved in cellular metabolism and induces significant cardiac dysfunction (2). HCM is an autosomal dominant genetic disease that is characterized by abnormal hypertrophy and deformation of the myocardial cells, abnormal arrangement of the myocardial fibers, and interstitial fibrosis (3). The overall survival of patients with cardiac amyloidosis is 4–8 months (4–6). On the other hand, HCM causes sudden death in youth and young adults (7). Differential diagnosis of these two cardiomyopathies is difficult because of similar clinical manifestations. The misdiagnosis rate of cardiac amyloidosis is as high as 35% (1). Therefore, it is important to accurately discriminate between the two types of myocardial lesions to determine timely clinical interventions that can improve survival rates.

Cardiac MRI (CMRI) is a highly specific and sensitive method for the clinical diagnosis of AL-CA. Myocardial strain is a functional CMRI index that estimates the shortening, lengthening, or thickening of the myocardium through a cardiac cycle. Therefore, cardiac magnetic resonance feature tracking (CMR-FT) analysis can be used to estimate the longitudinal, circumferential, and radial myocardial strains from the cine sequence images and determine the systolic and diastolic functions of the myocardium (8). Moreover, this method does not require injection of a contrast agent and can be used to study the severity of various myocardial diseases, especially in patients with renal insufficiency. LV global longitudinal strain (GLS) is a useful clinical parameter to determine subclinical cardiac dysfunction and an independent predictor of cardiovascular events (9–11). Recent studies have shown that global circumferential strain (GCS) can be used for the clinical evaluation of myocardial dysfunction (12). However, post-processing of global strain is time-consuming and complex. Furthermore, the algorithms used by different manufacturers to estimate global strains require further standardization. Therefore, their clinical application is limited. Riffel et al. (13) reported that the LV long-axis strain (LAS) was an alternative, simple, and feasible parameter that can be quickly and easily acquired to determine the longitudinal contractile function of the left ventricle in subjects with cardiac dysfunction. In this study, we evaluated the diagnostic performances of LV-LAS and other global peak systolic strains derived from CMR-FT in discriminating between AL-CA and HCM patients.

## Materials and methods

### Study population

This retrospective study was approved by the Ethical Review Committee of Qilu Hospital and was exempted from the requirement of providing an informed consent. This study enrolled 89 participants, aged between 38 and 75 years, which consisted of 30 AL-CA patients, 30 HCM patients, and 29 age-matched healthy controls. The inclusion criteria were based on the position statement of the European Society of Cardiology (ESC) working group (14). The pathological deposition of amyloid was confirmed using abdominal subcutaneous fat biopsy or endomyocardial biopsy. The diagnostic imaging features of AL-CA included left ventricular hypertrophy (with thickness of ≥13 mm) with intramyocardial granular echo on an echocardiography and delayed enhancement characteristics on the cardiac magnetic resonance (CMR) images. All the patients underwent CMRI acquisitions a week before the biopsy. HCM was defined as an unexplained LV hypertrophy with a maximal LV wall thickness of ≥15 mm or ≥13 mm for members with a family history of HCM. However, patients with thickening of the left ventricular wall due to increased load or other reasons and those with obstructive HCM were excluded. Extracardiac features, family history, and genotype were integrated in some patients for an accurate and informed diagnosis by experienced clinicians. In all the study subjects, the glomerular filtration rate was higher than 30 ml/min. The study subjects did not have any incompatible devices. Patients with chronic diseases such as cardiovascular disease, diabetes, hypertension (>140/90 mmHg), and a family history of arrhythmia were excluded.

### CMR protocol

MRI acquisitions were performed using a GE Signa HDx 3.0T scanner (GE Healthcare, USA) equipped with a 16-channel phased-array cardiac coil. The patients were placed in a supine position during the acquisition. The CMRI scan protocol included cine imaging and late gadolinium enhancement (LGE) imaging. Stacks of steady-state free precession (SSFP) end-expiratory breath hold cine images were acquired for the two-chamber and four-chamber views of the long-axis and 9–11 short-axis slices that covered the entire left ventricle from the mitral annulus to the apical epicardium. The typical cine imaging parameters were as follows: repetition time (TR)/echo time (TE), 10 ms/1.4 ms; flip angle (FA), 45°; matrix, 256 × 224; field of view (FOV), 350–400 mm; and slice thickness, 8 mm. Ten to fifteen minutes after an intravenous bolus administration of 0.2 mmol/kg Gd-DTPA (Magnevist, Bayer, Berlin, Germany), myocardial LGE images were acquired with a 2D phase-sensitive inversion recovery (PSIR) gradient-echo pulse sequence. The long-axis and short-axis view images were acquired at the same positions as acquired for the cine images. The parameters of the LGE imaging 2D PSIR gradient-echo pulse sequencing were as follows: TR/TE/FA, 2 R–R intervals/3.1 ms/15°; matrix, 256 × 224; FOV, 350–400 mm; and slice thickness, 8 mm.

### CMR imaging analyses

Two experienced radiologists with >5 years of experience analyzed the SSFP sequence images using the cvi42 software (version 5, Circle Cardiovascular Imaging Inc., Calgary, Canada). The phase of maximal and minimal volume of the left ventricle was defined as end diastolic and end systolic, respectively. The left ventricular endocardial and epicardial contours were delineated semi-automatically. The parameters of the left ventricular cardiac function were automatically generated by software. The papillary muscles and ventricular trabeculae were excluded from the myocardial mass but were included in the ventricular volume. The parameters of the general LV CMR imaging that were estimated included the left ventricular end-diastolic volume index (LVEDVI), left ventricular end-systolic volume index (LVESVI), left ventricular ejection fraction (LVEF), wall thickness maximum (WTMax), and left ventricular myocardial mass index (LVMI).

The left ventricular LAS was defined as the percentage of LV longitudinal axis shortening between the end-diastolic and end-systolic phases. The distance between the epicardial border of the LV apex and the center of a line that connects the origins of the mitral valve leaflets was measured in both the end-systolic and end-diastolic phases and defined as *length_end_syst_* and *length_end_diast_*, respectively. The percentage of LAS was then determined according to the following strain formula:

$$\mathrm{LAS}(\text{\%})=\frac{length_{end\_syst}-length_{end\_diast}}{length_{end\_diast}}*100$$

**Figure 1:**
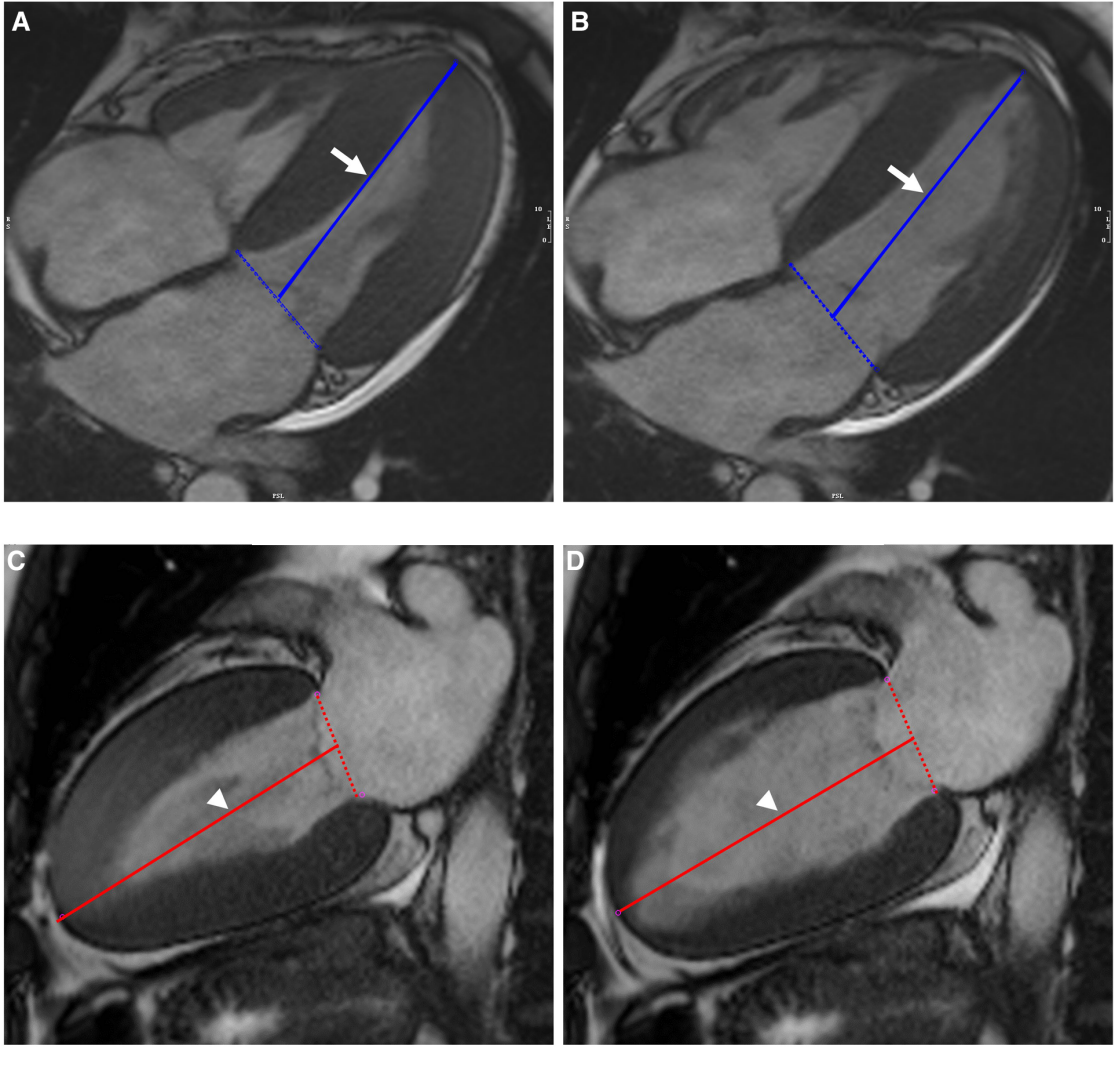
Illustration of biplanar assessment of LV LAS in an AL-CA patient on MR cine images: the solid blue line on end-systole (**A**) and end-diastole (**B**) of four-chamber view represented the distance between the epicardial border of the LV apex and the middle of a line connecting the origins of the mitral valves (white arrow). LV LAS was defined as the percentage in longitudinal shortening of the LV between end-diastole and end-systole. The measurements were performed on end-systole (**C**) and end-diastole (**D**) of two-chamber view and were shown as solid red line (white arrowhead). The average of the two LAS values was taken. LV, left ventricular; LAS, long-axis strain; AL-CA, light-chain patients with cardiac amyloidosis.

The average of the measurements in the two-chamber and four-chamber views was used for the final analyses (Figure 1).

The short-axis, two-chamber, four-chamber, and LV inflow–outflow tract views from the cine sequences were imported into the feature tracking module. The myocardial regions of interest (ROIs) were obtained by drawing the contours of the endocardium and the epicardium during the maximum diastolic phase. The organization tracking analysis was performed to obtain the strain curves and the bull's-eye charts of various global strain parameters such as GLS, GCS, and the global radial strain (GRS).

Myocardial LGE was classified into three patterns. The percentages of the three enhancement modes in the two patient groups (AL-CA and HCM) were calculated and compared. The LGE pattern was described as subendocardial if the delayed enhancement was observed only in the subendocardial layer. The LGE pattern was described as transmural if the delayed enhancement pattern was circumferential and involved the complete subendocardial layer through to the epicardium. The focal patchy LGE was limited to one or several separate segments of the myocardium.

### Intra- and inter-observer reproducibility assessments of LV strains

The random stratified sampling method was used to measure LV strains. Intra-observer reliability was assessed when the same investigator performed a masked review of 30 randomly selected CMRI examinations. Independently and in a blinded manner, inter-observer reliability was assessed when two investigators reviewed 30 randomly selected CMRI examinations. Both intra- and inter-observer reliability were estimated for LV-LAS, GRS, GCS, and GLS.

### Statistical analysis

Statistical analysis was performed using the SPSS Statistics software version 23.0 (SPSS Inc., International Business Machines, Chicago, IL, United States). The continuous variables (measurement data) were presented as means ± SD. The categorical data were expressed as percentages. The quantitative data between groups were compared using the analysis of variance (ANOVA), and the LSD test was used for the pairwise comparisons of the means. Chi-square and Fisher’s exact tests were used to compare the categorical data between different patient groups. *P* < 0.05 was considered as statistically significant. Spearman's correlation analysis was used to evaluate relationships between the ordinal variables. Receiver operating characteristic (ROC) curves were used to evaluate the differential diagnosis between AL-CA and HCM for each parameter. The values of the area under the ROC curve (AUC) were calculated, and the best cut-off values were estimated according to the Youden index. *P *< 0.05 was considered as statistically significant. The AUC values of ≥0.70 were considered adequate. The AUC values between 0.80 and 1.00 were considered good-to-excellent diagnostic efficacy (15). Interclass correlation coefficients (ICCs) were used to assess the intra- and inter-observer reproducibility of the CMR-derived global strain parameters such as GRS, GCS, and GLS. Intra- and inter-observer reproducibility of LV-LAS was assessed with both ICC (ICC_intra and ICC_inter) and Bland–Altman plots. ICCs were defined as excellent (ICC ≥ 0.75), good (ICC = 0.60–0.74), moderate (ICC = 0.40–0.59), or poor (ICC ≤ 0.39) (16).

## Results

### Baseline characteristics and general CMR parameters

Table 1 shows the baseline characteristics and general CMR parameters of the AL-CA patients, the HCM patients, and the control group. The AL-CA patients, the HCM patients, and the normal controls did not show any significant differences in age (*P *= 0.925), gender (*P *= 0.396), and body surface area (BSA) (*P *= 0.085). The heart rate of the AL-CA patients was significantly higher than that of the subjects in the other two groups (*P *= 0.012). The systolic blood pressure (SBP) and the diastolic blood pressure (DBP) of the AL-CA patients were significantly lower than those of the subjects in the other two groups (both *P *< 0.001). Patients with AL-CA showed significantly lower LVEF and higher LVESVI and LVMI compared to the HCM patients and healthy controls (all *P *< 0.001). There were no significant differences in the LVEDVI values between the three groups (*P *= 0.057). Furthermore, there were no significant differences in the WTMax values between the AL-CA and HCM patients (*P *= 0.927). Moreover, WTMax values of the two patient groups were significantly higher than those of the healthy controls (*P *< 0.001).

**Table 1:** Baseline characteristics and general CMR parameters of AL-CA patients, HCM patients, and healthy controls.

	AL-CA (*n *= 30)	HCM (*n *= 30)	Control (*n *= 29)	*P-*value (AL-CA vs*.* HCM)	*P-*value (all groups)
Male gender (%)	23 (76.7)	22 (73.3)	21 (72.4)	0.766	0.925
Age (year)	57 ± 10	54 ± 7	56 ± 7	0.182	0.396
BSA (m^2^)	1.71 ± 0.36	1.86 ± 0.22	1.81 ± 0.10	0.030	0.085
Heart rate (beat/min)	76 ± 10	70 ± 15	67 ± 10	0.057	0.012
SBP (mmHg)	108 ± 17	124 ± 12	120 ± 6	<0.001	<0.001
DBP (mmHg)	69 ± 11	79 ± 9	80 ± 5	<0.001	<0.001
LVEF (%)	44.3 ± 10.7	64.1 ± 7.9	63.1 ± 5.5	<0.001	<0.001
LVEDVI (ml/m^2^)	77.4 ± 19.5	80.4 ± 21.1	69.4 ± 10.9	0.519	0.057
LVESVI (ml/m^2^)	42.4 ± 13.3	29.1 ± 11.9	26.0 ± 4.9	<0.001	<0.001
WTMax (mm)	16.9 ± 2.7	16.9 ± 3.5	9.5 ± 1.7	0.927	<0.001
LVMI (g/m^2^)	84.7 ± 23.4	73.6 ± 18.4	45.1 ± 6.7	0.019	<0.001

AL-CA, light-chain cardiac amyloidosis; HCM, hypertrophic cardiomyopathy; BSA, body surface area; SBP, systolic blood pressure; DBP, diastolic blood pressure; LVEF, left ventricular ejection fraction; LVEDVI, left ventricular end-diastolic volume index; LVESVI, left ventricular end-systolic volume index; WTMax, wall thickness maximum; LVMI, left ventricular myocardial mass index.

*P* < 0.05 indicates statistically significant difference.

The values are presented as mean ± standard deviation or number (percentages).

**Figure 2:**
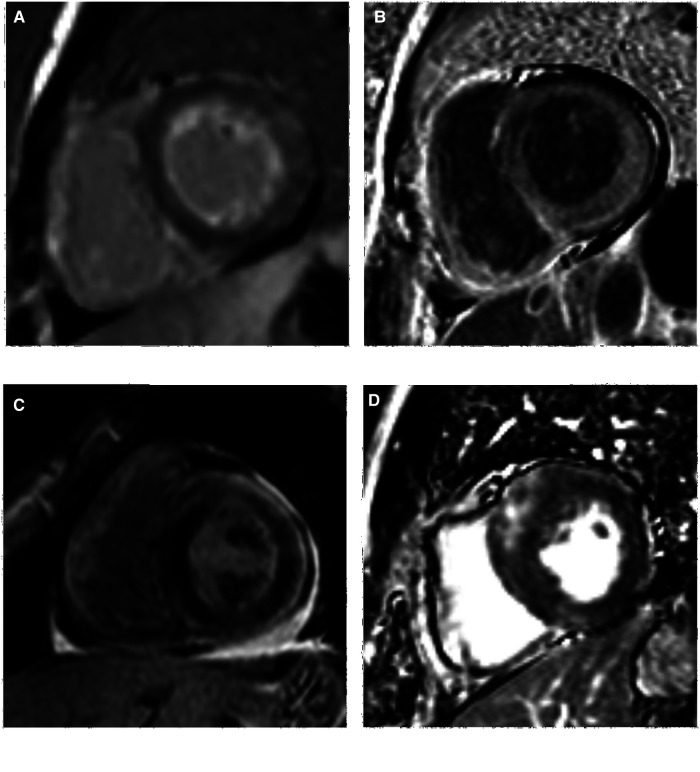
LV LGE images of AL-CA and HCM patients. (**A,B**) subendocardial and transmural LGE patterns of AL-CA patient, respectively; (**C,D**) focal patchy LGE pattern of AL-CA and HCM patient, respectively. LV, left ventricular; LGE, late gadolinium enhancement; AL-CA, light-chain patients with cardiac amyloidosis; HCM, hypertrophic cardiomyopathy.

Delayed scanning demonstrated LV myocardial LGE in all the AL-CA patients (30 cases, 100%) with subendocardial, transmural, and focal patchy patterns in 15 (50%), 12 (40%), and 3 (10%) AL-CA patients, respectively (Table 2). Among the 30 HCM patients, 24 (80%) of them showed a focal patchy LGE pattern, but the remaining 6 (20%) did not show any myocardial LGE (Figure 2). These data showed significant differences in the percentages of the three LGE patterns between the AL-CA and HCM patient groups. LV myocardial LGE was not detected in any of the 29 healthy controls. Therefore, LV myocardial LGE patterns of the two patient groups were significantly different compared to the healthy controls (all *P *< 0.001).

**Table 2:** Comparison of LV myocardial LGE pattern of AL-CA patients, HCM patients, and healthy controls.

LGE pattern	AL-CA (*n *= 30)	HCM (*n *= 30)	Control (*n *= 29)	*P-*value (AL-CA vs*.* HCM)	*P-*value (all groups)
Subendocardial LGE, *n* (%)	15 (50)	0	0	<0.001	<0.001
Transmural LGE, *n* (%)	12 (40)	0	0	<0.001	<0.001
Focal patchy LGE, *n* (%)	3 (10)	24 (80)	0	<0.001	<0.001
Non-LGE, *n* (%)	0	6 (20)	29 (100)	<0.001	<0.001

AL-CA, light-chain cardiac amyloidosis; HCM, hypertrophic cardiomyopathy, LGE, late gadolinium enhancement.

*P* < 0.05 indicates statistically significant difference.

The values are presented as number (percentages).

### Intra- and inter-observer reproducibility assessment for LV strain parameters

**Figure 3:**
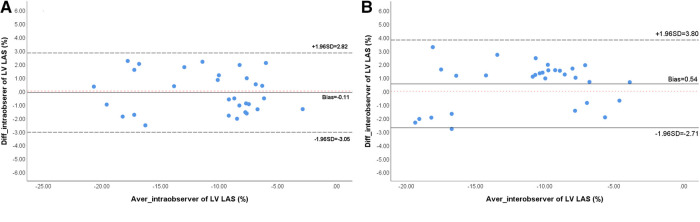
Bland-Altman analyses of intra-observer (**A**) and inter-observer (**B**) reproducibility for LV-LAS. LV, left ventricular; LAS, long-axis strain.

Both the intra-observer (ICC = 0.953; 95% CI, 0.904–0.978) and inter-observer (ICC = 0.932; 95% CI, 0.859–0.967) measurements of LV-LAS showed excellent reliability. The Bland–Altman plots are shown in Figure 3. CMRI-derived strain parameters also showed excellent reproducibility. The intra-observer ICC values for the GRS, GCS, and GLS measurements were 0.941 (95% CI, 0.845–0.974), 0.949 (95% CI, 0.898–0.975), and 0.965 (95% CI, 0.909–0.985), respectively. The inter-observer ICC values for the GRS, GCS, and GLS measurements were 0.914 (95% CI, 0.832–0.957), 0.907 (95% CI, 0.818–0.953), and 0.911 (95% CI, 0.826–0.955), respectively (Table 3).

**Table 3:** Intra- and inter-observer reproducibility of CMR LV strain parameters.

	GRS		GCS		GLS		LAS	
	Intra	Inter	Intra	Inter	Intra	Inter	Intra	Inter
ICC	0.941	0.914	0.949	0.907	0.965	0.911	0.953	0.932
Lower CI	0.845	0.832	0.898	0.818	0.909	0.826	0.904	0.859
Upper CI	0.974	0.957	0.975	0.953	0.985	0.955	0.978	0.967

CMR, cardiovascular magnetic resonance; LV, left ventricular; CI, confidence interval; GRS, global radial strain; GCS, global circumferential strain; GLS, global longitudinal strain; LAS, long-axis strain.

### LV-LAS and LV global strain parameters analysis

**Figure 4:**
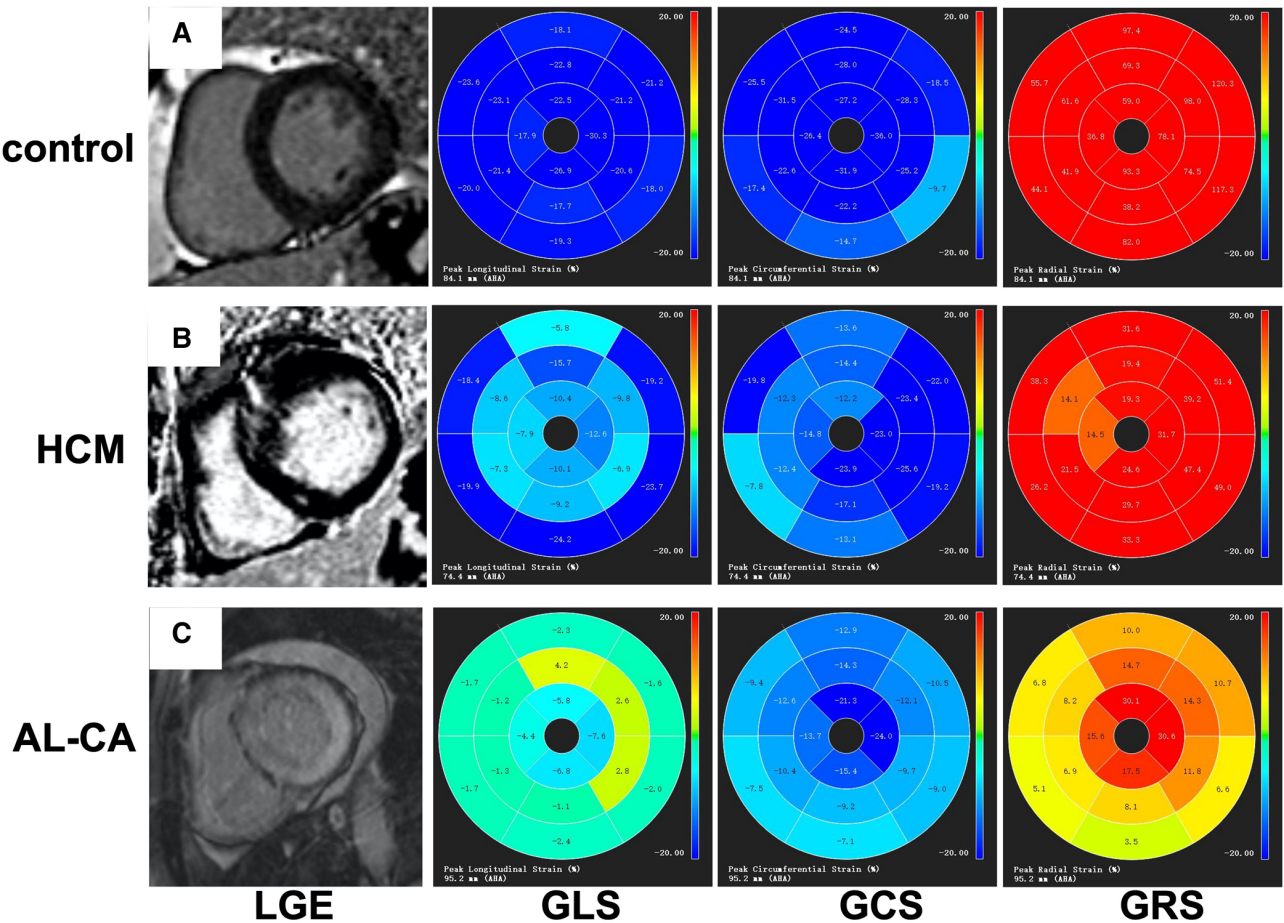
Examples of LV LGE images and bull's-eye charts of global strain. (**A**) A healthy control with negative LGE and normal value of strains. (**B**) A HCM patient with focal patchy LGE; segmental strain abnormalities were seen. (**C**) An AL-CA patient with transmural LGE and diffused abnormal global strains. LV, left ventricular; LGE, late gadolinium enhancement; GRS, global radial strain; GCS, global circumferential strain; GLS, global longitudinal strain; HCM, hypertrophic cardiomyopathy; AL-CA, light-chain patients with cardiac amyloidosis.

The values of LV strain parameters for the AL-CA patients, the HCM patients, and the healthy controls are shown in Table 4. The left ventricular-LAS, GLS, and GCS values were significantly higher in the AL-CA patients compared to the HCM patients and the healthy controls (all *P *< 0.001). Furthermore, GRS values of the AL-CA patients were significantly lower than those of the other two groups (all *P *< 0.01). The LAS, GLS, and GCS values of the AL-CA patients were significantly higher than those of the HCM patients (all *P *< 0.001). The GRS value of the AL-CA patients was significantly lower than that of the HCM patients (*P *< 0.001). Figure 4 shows the LGE images and the LV global strain values from a representative healthy control, a HCM patient, and an AL-CA patient.

**Table 4:** Comparison of LV strain parameters of AL-CA patients, HCM patients, and healthy controls.

CMR strain	AL-CA (*n* = 30)	HCM (*n* = 30)	Control (*n* = 29)	*P-*value (AL-CA vs*.* HCM)	*P-*value (all groups)
LAS (%)	−5.1 ± 2.4	−11.5 ± 3.6	−17.2 ± 2.7	<0.001	<0.001
GLS (%)	−8.6 ± 2.6	−12.4 ± 3.1	−15.9 ± 3.6	<0.001	<0.001
GCS (%)	−12.2 ± 2.9	−17.8 ± 2.6	−20.5 ± 2.4	<0.001	<0.001
GRS (%)	17.7 ± 5.9	31.2 ± 7.1	37.7 ± 8.4	<0.001	<0.01

AL-CA, light-chain cardiac amyloidosis; HCM, hypertrophic cardiomyopathy; CMR, cardiac magnetic resonance; LAS, long-axis strain; GLS, global longitudinal strain; GCS, global circumferential strain; GRS, global radial strain.

*P* < 0.05 indicates statistically significant difference.

### ROC curves and AUC analysis of general CMR parameters and strains

Among the myocardial morphological and functional parameters, LVEF showed an excellent diagnostic performance in discriminating AL-CA from HCM (AUC = 0.927, *P* < 0.001). The best cut-off value for LVEF was 43.5% with a sensitivity of 82.8% and a specificity of 86.2%. LVESVI showed a good diagnostic performance in discriminating AL-CA from HCM (AUC = 0.826, *P* < 0.001). The best cut-off value for LVESVI was 31.995 ml/m^2^ with a sensitivity of 86.2% and a specificity of 72.4%. The routine CMR parameters such as LVMI (AUC = 0.631, *P* = 0.086), WTMax (AUC = 0.533, *P* = 0.663), and LVEDVI (AUC = 0.446, *P* = 0.479) did not show any significant diagnostic efficacy in discriminating AL-CA from HCM. The Supplementary Table illustrated the sensitivity, specificity, Youden index, and cut-off value of CMR parameters with AUC values of ≥0.7 for AL-CA diagnosis.

**Figure 5:**
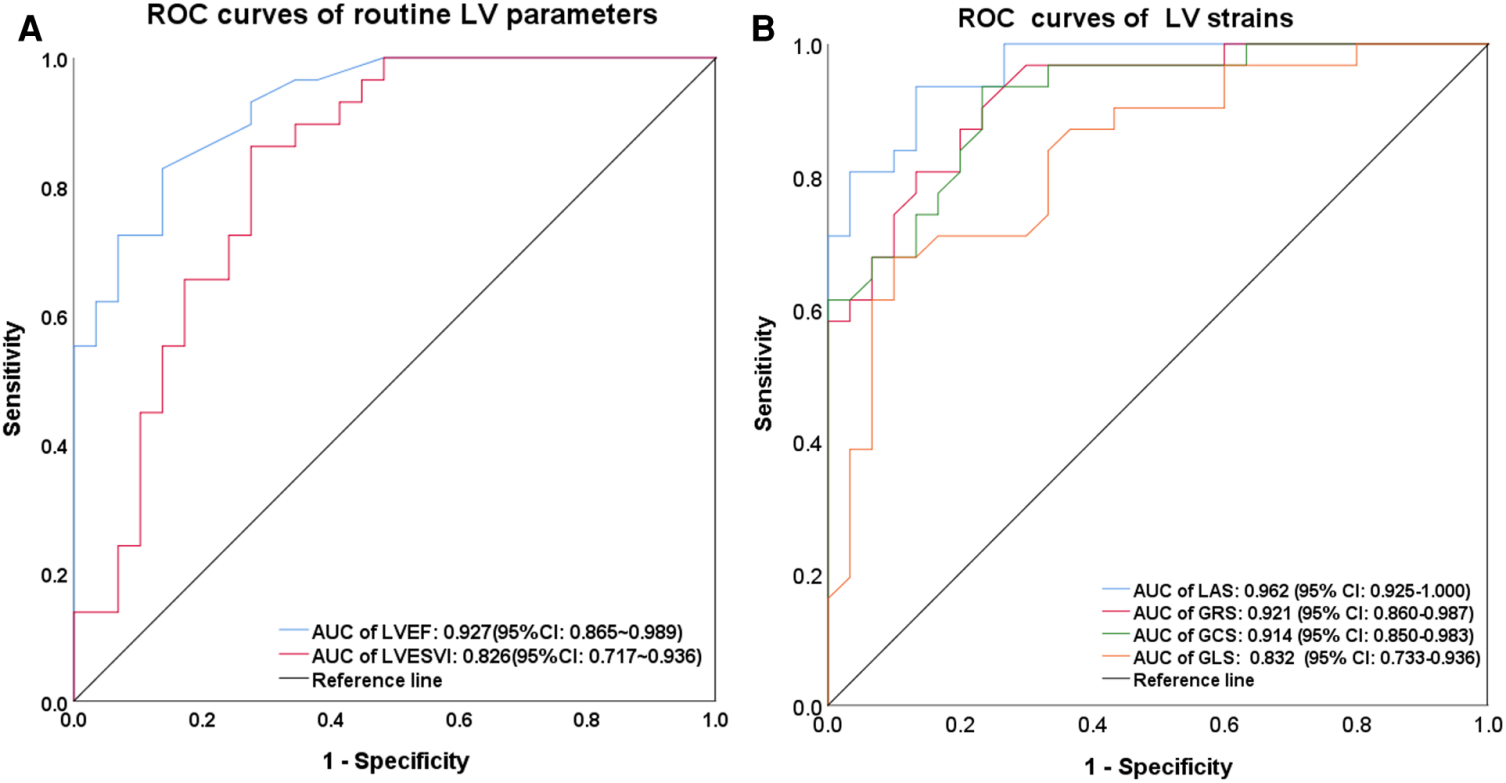
ROC curves in discriminating AL-CA from HCM. In ROC curve of routine left ventricular parameters (**A**), LVEF and LVESVI had a significant differential diagnostic efficacy, with the AUC of 0.927(*P *< 0.001) and 0.826 (*P *< 0.001), respectively. In ROC curve of left ventricular strain parameters (**B**), LAS demonstrated the highest AUC of 0.962 (*P* < 0.001) and 0.921 (*P* < 0.001) for GRS, 0.914 (*P* < 0.001) for GCS, and 0.832 (*P* < 0.001) for GLS, respectively. ROC, receiver operating characteristic; LV, left ventricular; LVESVI, left ventricular end-systolic volume index; AUC, area under the curve; LAS, long-axis strain; GRS, global radial strain; GCS, global circumferential strain; GLS, global longitudinal strain; HCM, hypertrophic cardiomyopathy; AL-CA, light-chain patients with cardiac amyloidosis.

All the strain parameters showed good or excellent diagnostic performances in discriminating between AL-CA and HCM. Among the strain parameters, LAS showed the best diagnostic efficacy in discriminating AL-CA from HCM (AUC = 0.962, *P* < 0.001). The best cut-off value for LAS was −8.435% with sensitivity of 93.3% and specificity of 86.7%. In terms of diagnostic efficacy, LAS was followed by GRS (AUC = 0.921, *P *< 0.001), GCS (AUC = 0.914, *P* < 0.001), and GLS (AUC = 0.832, *P* < 0.001). The best cut-off values for GRS, GCS, and GLS were 23.00% (sensitivity, 80.6%; specificity, 86.7%), −16.05% (sensitivity, 93.3%; specificity, 76.7%), and −9.3% (sensitivity, 63.3%; specificity, 93.3%), respectively. The ROC curves for myocardial strain and morphological parameters with AUC values of ≥0.7 are shown in Figure 5.

### Correlation analysis of LV-LAS and global strain parameters

**Figure 6:**
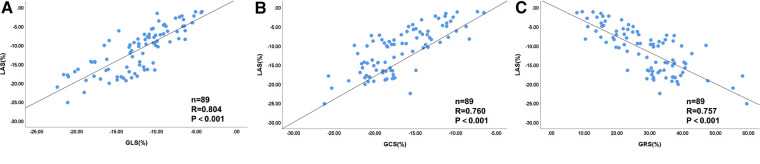
Scatter diagrams of correlation analysis between LV LAS and global strains for all patients (*n *= 89). The correlation coefficient was as follows: (**A**) LAS-GLS (*R* = 0.804, *P *< 0.001), (**B**) LAS-GCS (*R* = 0.760, *P* < 0.001), and (**C**) LAS-GRS (*R* = −0.757, *P* < 0.001). LV, left ventricular; GLS, global longitudinal strain; GCS, global circumferential strain; GRS, global radial strain.

Spearman's correlation analysis was performed to determine the relationship between LAS and the three directional global strains. We observed highly significant positive correlation between LAS and GLS (*R* = 0.804, *P *< 0.001), moderate positive correlation between LAS and GCS (*R* = 0.760, *P* < 0.001), and moderate negative correlation between LAS and GRS (*R* = −0.757, *P* < 0.001). The scatter diagrams of Spearman's correlation analysis are shown in Figure 6.

## Discussion

The diagnostic value of CMRI is well established and widely accepted in cardiomyopathy, and LVEF is most commonly used to measure the cardiac systolic function (17, 18). LVEF can measure changes in the left ventricular volume but cannot estimate changes in the myocardial contractility or changes in the myocardial segmental movements. Zerhouni et al. used CMRI to measure myocardial strain, a parameter for estimating cardiac functional parameters that cannot be evaluated using LVEF (19). Myocardial strain represents deformation (shortening, lengthening, or thickening) of the myocardium under tension, and changes in the myocardial length during the cardiac cycle are expressed as the percentage of the original myocardial length (20). Therefore, CMRI can be used to quantify the global and regional myocardial wall motions and measure the myocardial longitudinal, radial, and circumferential strains (21). CMR-FT is an emerging method for estimating the degree of myocardial deformation (22). The displacement of myocardial segments can be estimated by analyzing the conventional SSFP cine sequence images without additional scanning.

Since CMRI-derived strain parameters show a potential clinical value in disease diagnosis and prognosis stratification, the reproducibility of data is important. Several studies have evaluated the reproducibility of data from CMR-FT and shown that the values for various global strain parameters from CMR-FT are highly reproducible and accurate (23–25). Bucius et al. (24) assessed the reproducibility of global strains such as GLS and GCS, and segmental strains such as segmental longitudinal (SLS) and segmental circumferential (SCS) were derived from CMR-FT. The intra- and inter-observer reproducibility were excellent for both GLS (ICC: 0.95, 0.92) and GCS (ICC: 0.89, 0.86), respectively. In the reproducibility assessment of the segmental strain, the combined intra-observer agreement was excellent in both SLS (ICC = 0.914, *P* < 0.001) and SCS (ICC = 0.885, *P* < 0.001). Schmidt et al. (26) investigated the reproducibility of global strain and strain rate (SR) parameters in 20 healthy subjects and 20 patients with acute myocarditis and showed excellent intra-observer reproducibility for most global LV strains and SR parameters (range of ICC: 0.81–1.00) with the exception of only global radial SR, which showed poor reproducibility (ICC: 0.23). Furthermore, the inter-observer reproducibility for all the LV strain and SR parameters was slightly lower than the intra-observer reproducibility. However, they still showed good-to-excellent reproducibility for the longitudinal and circumferential strain and SR parameters (range of ICCs: 0.66–0.93). In our study, the repeatability of GCS and GLS was excellent for both the intra-observer measurements (ICC: 0.949–0.965) and the inter-observer measurements (ICC: 0.907–0.911). Moreover, the intra- and inter-observer reproducibility of GRS were excellent (ICC: 0.941–0.914) with a high AUC value (AUC 0.921). This suggested that GRS was highly reproducible and consistent using a post-processing software and semi-automatic analysis. Further studies are necessary to validate the reliability and diagnostic efficacy of GRS.

LGE is a parameter that is commonly used for myocardial tissue characterization and can be used to estimate the extent of an amyloid burden in the myocardium. Compared to the endomyocardial biopsy, Brownrigg et al. showed that the sensitivity and specificity values of CMRI in diagnosing cardiac amyloidosis based on LGE were 85.7% and 92%, respectively (27). The subendocardial and transmural patterns of LGE are used for the differential diagnosis of cardiac amyloidosis. Dohy et al. (28) reported that the sensitivity and specificity values for the diffuse septal subendocardial LGE in the diagnosis of cardiac AL amyloidosis were 88% and 100%, respectively. Several studies have shown that the abnormal myocardial strain in patients with cardiac amyloidosis is closely related to the LGE patterns. Erley et al. (29) analyzed the reproducibility of CMR-derived GLS and GCS and the correlation between strain parameters and LGE in 50 patients. Repeated measurements showed low intra- and inter-observer variability for both GLS and GCS (ICCs, 0.86–0.99; coefficients of variation, 3%–13%). The study also showed that both GLS and GCS were associated with LGE, and the association between LGE and GCS was significantly higher (AUC 0.77–0.78) than the correlation between LGE and GLS (AUC 0.67–0.72).

Oda et al. (30) analyzed the relationship between GCS and LGE in 61 patients with myocardial amyloidosis and showed that the GCS value was significantly lower in the LGE-positive patients than that in the LGE-negative patients (*P *< 0.01). The sensitivity of GCS in the detection of LGE-positive amyloidosis was high (93.8%) and suggested that GCS could be used to accurately detect the severity of cardiac amyloid burden. In this study, the GCS value of the AL-CA patients was significantly lower than that of the HCM patients and the healthy controls (both *P *< 0.001). This suggested that the left ventricular damage in the myocardium of the AL-CA patients was significantly higher than that of the HCM patients. Moreover, ROC curve analyses of the myocardial strain indexes for the differential diagnosis of HCM and AL-CA showed that the AUC value for GCS was higher than the AUC value for GLS (0.914 vs. 0.832). This further suggested that GCS was a highly accurate and more sensitive parameter for discriminating between HCM and AL-CA.

LAS is a new index of left ventricular systolic function that can be easily measured from the CMR cine images with high reproducibility (31). In this study, we showed that the estimates of LAS were highly reliable and consistent in both intra- and inter-observer measurements (ICCs: 0.932–0.953). Recent studies have reported a close relationship between LAS and the feature tracking-derived longitudinal strain (32, 33). A series of images for the left ventricular long-axis, short-axis, and outflow tract are required for the conventional GLS measurements. However, many seriously ill patients cannot undergo all the MRI scanning sequences. LAS can be measured from the end-diastolic and end-systolic phases of the long-axis cine images along the two-chamber and four-chamber views. In comparison with the global longitudinal strain, LAS measures the LV longitudinal shortening between the end-diastole and the end-systole, but does not provide the strain trend or the strain curve for the whole cardiac cycle. However, LAS can be easily acquired in a shorter period of time and is therefore amenable for seriously ill patients. Leng et al. analyzed patients with heart failure (34) and showed that LAS was better than the conventional strain parameters in the differential diagnosis between the patients with heart failure with preserved ejection fraction (HFpEF) and the healthy controls. Therefore, LAS is an effective surrogate measure of GLS. Riffel et al. estimated LAS values from 234 selected healthy volunteers and showed that the average reference LAS value was −17.1% ± 2.3% (13). The LAS value from the healthy controls in our study was consistent with that reported by Riffel et al. (−17.2% ± 2.7%). Our data also showed a significant correlation between LAS and GLS (*R* = 0.804). Furthermore, the LAS values of HCM and AL-CA patients were significantly lower than those of the healthy controls, and the LAS values of the AL-CA patients were significantly lower than those of the HCM patients. Besides, ROC curve analysis showed that the AUC value of LAS (0.962) was the highest among all the parameters. These results demonstrated the potential clinical utility of LAS in discriminating AL-CA patients from HCM patients.

This study has some limitations. First, this was a single-center study with a small sample size that may have resulted in bias. Therefore, future large-cohort multicenter studies are required to confirm our findings. Second, although both systolic and diastolic strain parameters were analyzed during post-processing of CMRI, only systolic strain parameters were analyzed in this study. Therefore, we plan to analyze the diastolic strain data in the future studies. Finally, the relationship between the strain parameters and LGE was not assessed in this study and needs to be analyzed in the future.

In conclusion, our study demonstrated that LAS was a simple and reliable index of the LV systolic functional status in the AL-CA patients and could accurately distinguish them from the HCM patients. Furthermore, CMR-derived global peak systolic strains showed a significant potential in evaluating the myocardial systolic function in patients with contraindications to gadolinium-based contrast agents.

## Data Availability

The raw data supporting the conclusions of this article will be made available by the authors, without undue reservation.
