# Supplementary material for: CMR left ventricular strains beyond global longitudinal strain in differentiating light-chain cardiac amyloidosis from hypertrophic cardiomyopathy
Source: Front Cardiovasc Med. 2023 May 3;10:1108408. doi: 10.3389/fcvm.2023.1108408 (PMC10188937; doi:10.3389/fcvm.2023.1108408)
Supplement: Supplementary file 1 [file Datasheet1.pdf]

Supplementary table:

Sensitivity, specificity, Youden index and cut-off value of CMR parameters(AUC > 0.7) for AL-CA

	AUC	95%CI	Sensitivity	Specificity	Cut-off value	Youden Index
LVEF	0.927	0.865~0.989	0.828	0.862	43.5%	0.690
LVESVI	0.826	0.717~0.936	0.862	0.724	31.995ml/m^2	0.586
LAS	0.962	0.925~1.000	0.933	0.867	-8.435%	0.802
GRS	0.921	0.860~0.987	0.806	0.867	23.00%	0.673
GCS	0.914	0.850~0.983	0.933	0.767	-16.05%	0.702
GLS	0.832	0.733~0.936	0.633	0.933	-9.30%	0.577

CMR, cardiovascular magnetic resonance; AUC, area under the curve; AL-CA, light-chain cardiac amyloidosis; LVEF, left ventricular ejection fraction; LVESVI, left ventricular end-systolic volume index; LAS, long axis strain; GRS, global radial strain; GCS, global circumferential strain; GLS, global longitudinal strain.
